# Physicians’ Perspectives Regarding Private Equity Transactions in Outpatient Health Care—A Scoping Review and Qualitative Analysis

**DOI:** 10.3390/ijerph192315480

**Published:** 2022-11-22

**Authors:** Tim N. Nolte, Felix Miedaner, Sandra Sülz

**Affiliations:** 1 Erasmus School of Health Policy & Management, Erasmus University Rotterdam, Postbus 1738, 3000 DR Rotterdam, The Netherlands; 2 Faculty of Health and Health Care Sciences, Ostfalia University of Applied Sciences, Rothenfelder Str. 10, 38440 Wolfsburg, Germany

**Keywords:** private equity, outpatient health care, physician

## Abstract

Private equity (PE) investments in health care have increased drastically over the last decade, and the profit interests of these companies have triggered a vivid discussion among medical professions. However, what are the key underlying perceptions among physicians regarding this trend? Unravelling the argumentative structure of this debate is the purpose of this paper. With physicians being a major stakeholder group in the outpatient health care setting, this paper explores physicians’ perspectives regarding increasing PE activities. We systematically searched, selected, and synthesized existing knowledge in a scoping review and complemented the findings through 14 semi-structured interviews with physicians working in the outpatient health care sector in Germany. The results outline a complex network of arguments, concerns, and fears whereby the first intuitive perception of physicians is of critical nature. Arguments cluster around central perceptions of how PE involvement affects the individual autonomy of physicians in their daily work and decision-making, the impact on quality of care, work-life balance considerations, PE investment strategies, lack of medical vs. managerial expertise, and taxation issues. The high number of opinion papers among the literature underlines the actuality of the topic and emphasizes the need for empirical research.

## 1. Introduction

Private equity (PE) investments in health care have increased drastically, with an annual deal value rising from $41.5 billion in 2010 to $95.9 billion in 2020 globally [1]. This adds up to ‘approximately $750 billion over the last decade’ [1] (p. 4). In the United States (US), PE companies have been taking over large areas of outpatient health care companies because of the high growth potential associated with the highly fragmented market [2,3,4,5]. In the US market, Appelbaum and Batt [2] (p. 19) found that ‘between 2013 and 2019, 8 percent of PE investments were in hospitals compared to 30 percent in clinics and outpatient services.’ In the outpatient health care sector of the US, PE companies focus on high value ‘platform practices’, which describe the acquisition of large, medium, and small physician practices for expansion and consolidation [3,6]. Despite the huge differences between the US and the German health care systems, a similar trend can be observed in Germany, as the outpatient market provides comparable growth and revenue potentials for investors [7,8]. This trend is partly due to regulatory changes that allow non-medical investors to acquire, subject to legal requirements, health care facilities such as outpatient practices [7,8]. However, this acquisition trend is also fueled by the rising demand for health care facilities due to an aging population. Simultaneously, a consolidation trend is taking place in multiple health care systems around the world including the US and Germany, which stands in line with PE companies entering the market [2,7].

PE companies pursue a business model of growth equity investments in mature companies with the aim of returning profits [9,10,11]. These investment activities are financed by raising capital from limited partner investors (LPIs), such as large pension funds, corporations, and wealthy individuals who expect a higher return on investment than conventional investments and accept higher risks in return [2,5]. The LPIs’ capital flows into actively managed PE funds for which the PE company charges a fee [5,12]. Typically, PE funds consist of 98% LPI capital, and only 2% are provided by the partners and principals of the PE itself [2] (p. 6). These funds are then used to acquire (parts of) selected companies that will be subsequently managed through the PE company, either by appointing the company’s board of directors or by having the PE company’s principals take seats on the board of directors [2].

The profit interests of such PE companies have triggered a heated discussion among medical professionals in the US and Germany [2,6,8,11,13,14,15]. It is fiercely debated as to how far the PE’s profit interests undermine medical principles and negatively affect quality of care [11,13,14,15]. Sharp criticism is also expressed in light of the PE’s ‘buy-and-build’ strategy, i.e., the consolidation of small practices into a larger consortium followed by a resale [2] and the tendency to target wealthier areas with growth potential [8]. Zhu and Polsky [16] even compare this trend of increasing PE ownership in independent physician practices with the introduction of predators in different ecosystems. However, what is the core of the heated discussion? What arguments are brought up by physicians to support and criticise the trend of PE companies increasingly entering the outpatient health care sector? Unravelling and structuring this discussion will be the purpose of this paper. We examine emerging evidence, determine the scope of arguments, and contribute a systematic overview of physicians’ perspectives. This differentiated approach seeks to identify research opportunities and stimulate a more nuanced debate about PE involvement in outpatient health care.

## 2. Materials and Methods

Given the exploratory research objective, this study will combine a scoping review with semi-structured interviews. Scoping reviews are suitable for our purposes as they address exploratory research questions through systematically searching, selecting, and synthesizing existing knowledge [17]. By complementing the findings from the literature with semi-structured interviews, we can further study the perceptions and opinions of physicians regarding the arguments found in the previous scoping review and explore possible new arguments [18]. This form of qualitative data extraction enables an elaboration of meaningful issues to physicians and their perceptions towards PE companies increasingly buying outpatient physician practices [18]. This study was approved by the institutional ethics review committee (ETH2122-0535).

### 2.1. Scoping Review

#### 2.1.1. Identification of Relevant Literature for Scoping Review

To elaborate the medical perspectives on PE companies entering the outpatient physician practice market, the electronic databases PubMed and ScienceDirect were searched with a predefined search syntax [19]. This syntax consisted of ‘private equity’ to narrow the results to the exact organizational form. Subsequently, descriptors from the medical dimension, especially outpatient health care, were chosen, including: ‘healthcare’, ‘health care’, ‘ambulatory’, ‘outpatient’, ‘primary care’, ‘medical practic*’ and ‘physician practic*’ (with * being the wildcard character to match any number of literal characters).

Due to the research focus on the perceptions of physicians as well as the novelty of the research field, hand-searched practice-oriented literature was included. The inclusion of practice-oriented literature was considered necessary to identify current perceptions because scientific and peer-reviewed research literature is often delayed [19]. We focused on journals that aim for a wide audience of practising physicians, limited to a German-speaking audience, and from selected medical specialties that were commonly associated with PE investments. This set also included journals without a scientific peer-reviewing process to enable the inclusion of perceptions and opinions expressed in opinion papers, editorials, and commentaries etc. First, journals were selected based on the scope and the relevant medical specialties previously identified in the scientific literature. Additionally, the most popular medical journals for practising physicians in Germany were hand-searched [20]. Those included the following: ‘Der MKG-Chirurg’, ‘Deutsches Ärzteblatt’, ‘Arzt & Wirtschaft online’, ‘Ärzte Zeitung’, and ‘Medical Tribune’, which was considered suitable due to its medical-economic background. Second, these journals were manually searched for relevant articles focusing on PE companies entering the outpatient health care sector in Germany from the physicians’ perspective.

The literature search was performed between March and April 2021.

#### 2.1.2. Screening and Selection of Relevant Literature

To reflect the opinions and related arguments of physicians, we included original research and opinion papers consisting of research letters, editorials, and scientific commentaries. We included literature from the past 10 years (2011–2021) to maintain topicality. Furthermore, all medical specialties were included to provide an overview of the specialties most focused on by PE companies. A restriction by geographical region was not made, but articles had to be written in English or German. Articles were included if they reflected a medical perspective. This inclusion criterion was fulfilled if at least one author was affiliated with a medical institution. Literature was excluded if it did not focus on outpatient physician practices. After removing duplicates, the first author screened the results by title and abstract and subsequently assessed full-text articles for eligibility.

#### 2.1.3. Data Extraction

Each article was assessed to identify the central opinion and the related arguments used to support it. The opinions were categorized into supporting and criticizing arguments. Subsequently, common opinions in each category were clustered around related arguments. The data extracted from the results were thereby entered manually into the database program Microsoft Excel (Microsoft Corporation, Redmond, WA, USA) using a charting form in line with Arksey and O’Malley [21]. This ‘descriptive-analytical method within the narrative tradition, which involves applying a common analytical framework to all the primary research reports and collecting standard information on each study, stands more chance of being useful’ [21] (p. 26).

### 2.2. Semi-Structured Interviews

We conducted semi-structured interviews with self-employed and employed physicians in the outpatient healthcare sector in Germany to elaborate on the findings and arguments found in the scoping review.

#### 2.2.1. Participant Selection

The selection of interviewees was performed through criteria-focused sampling [22]. For the PE-relevant specialties identified in the literature (dentistry, oral and maxillofacial surgery, dermatology, ophthalmology, internal medicine), practising physicians were searched via the Google search engine. Overall, a total of 74 potential interviewees were contacted via email and the professional social network LinkedIn. Of these, 6 responded positively and the remaining 68 either refused or did not respond to a second request. Additionally, snowball sampling was used to identify additional interviewees (irrespective of specialty) at the end of each conducted interview. This resulted in 8 additional interviewees. Interviews were held with physicians from the medical fields of dentistry (*N* = 9), oral and maxillofacial surgery (*N* = 2), dermatology (*N* = 1), paediatrics (*N* = 1) and ophthalmology (*N* = 1). Out of the 14 interviewees, 12 were male and 2 were female. The mean age was 40.0 years (standard deviation: 12.4 years), and the mean practice time was 12.5 years (standard deviation: 10.6 years). Furthermore, 6 of the interviewees were self-employed, 8 were employed, and all were working in outpatient health care. Of all interviewees, 12 had a general understanding of PE companies and were aware of increasing PE involvement in their specialty. Four interviewees had contact with PE companies at the time of the interviews. A tabular overview of participant characteristics is provided in the Supplementary Material.

#### 2.2.2. Data Collection

All interviews were conducted by the first author (male). At the time of data collection, the first author was a student pursuing his M.Sc. graduation work with training in qualitative research methods as part of his Bachelor (Health Care Economics) and Master programme (Health Care Management). The interviewer did not have any relationship with the participants except for one participant (next of kin).

Prior to the interviews, the interviewees were informed about the purpose of the study, the interviewer’s study background, and the interview procedure. Interviews were structured through an interview guide to elaborate on all the major topics that were derived from the results of the scoping review [23]. Two practice interviews were conducted to pilot test the interview guide and its questions. The interview guide was not adjusted based on the pilot interview.

The interviews were confidential, with a duration of 27 to 64 min, and conducted in May and June 2021. Interviews were held and recorded using the online video communication platform Zoom allowing for audio and visual recording. In addition, the interviewer made field notes throughout the interview to document emotional expressions. In each instance, the participant and interviewer were both at home and no other people were present during the interview.

#### 2.2.3. Data Analysis

First, the interviews were transcribed into text format and returned to participants for comments and corrections. As this research focuses on perceptions, the transcripts included aspects of behaviour that were visible through the video format. Kowal and O’Connell [24] describe this as a key part of transcribing. Second, thematic analysis was used to study the transcripts and the coding was performed in stages by the first author. The first stage entailed detailed and intensive reading of the transcripts to identify similar arguments. In the next stage, the identified arguments were described in detail and put into a grid for the actual coding. The third stage included the actual coding. Schmidt [25] (p. 255) defines this as ‘relating particular passages in the text of an interview to one category, in the version that best fits these textual passages’. Thereby, the arguments were first roughly categorized into critical, supportive, and neutral arguments and subsequently clustered around themes. We used the findings from the scoping review to identify themes in advance, but we also allowed for new themes to emerge from the interview data. A similar data management structure to the scoping review was used (charting form Microsoft Excel, Microsoft Corporation, Redmond, WA, USA) and the final coding tree is provided in the Supplementary Material. Participants did not provide feedback on the findings.

## 3. Results

### 3.1. Descriptive Overview of Literature Results

**Figure 1:**
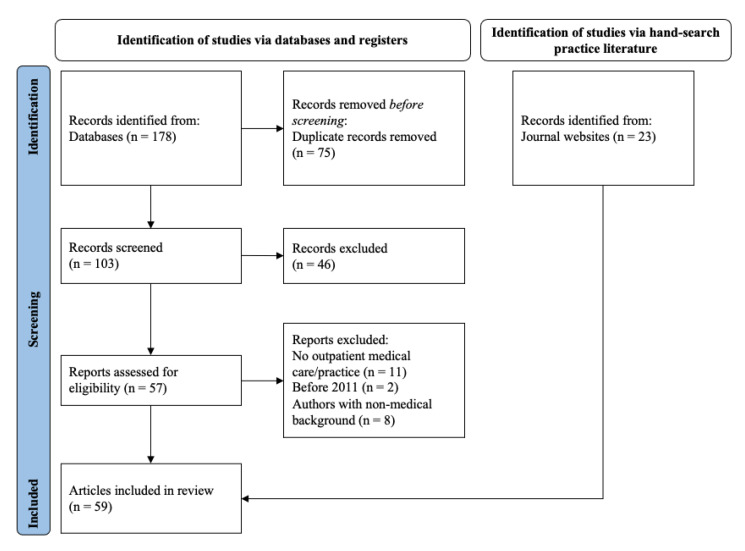
Flow diagram of the scoping review.

Figure 1 illustrates the screening process following the PRISMA guidelines [26]. In total, 59 articles were included in the qualitative synthesis. All relevant results were published between 2017 and 2021. The geographical background of the scientific literature was predominantly from the US, with all results having at least one author from the US. Of the 36 scientific articles, 75% were opinion papers such as editorials, viewpoints in scientific journals, or scientific commentaries.

### 3.2. Identified Perceptions and Arguments

Although identified perceptions and arguments regarding PE involvement in outpatient care are characterized by complex interdependencies, central perceptions can be mainly clustered into the impact of PE activities on the individual autonomy of physicians (1), quality of care (QoC) (2), work-life balance (3), and sustainability (4). In addition, the lack of medical vs. managerial expertise (5) and taxation issues (6) were identified as essential arguments.

#### 3.2.1. Impact on Physician Autonomy

The general tone in the literature reviewed was of a critical nature related to concerns about the demise of professional medical and self-employed managerial autonomy, and decision-making power being detached from physicians with PE involvement [27,28,29,30,31,32,33,34,35,36,37]. Correspondingly, Novice et al. [36] found that physician autonomy was the most common concern expressed by 81% of 137 residents from dermatology residency programs in the US. The concerns about physician autonomy are predominantly based on the belief that the PE business model conflicts with medical ideals by putting profits over patients [38]. It is commonly argued that the profit-oriented motivation of PE companies results in medical decisions being influenced away from patient wellbeing, as medical decision-making becomes detached from medical interests in favor of financial interests [4,11,37,39,40]. This concern is echoed in the interviews:

*I would no longer be free to make my own decision afterwards, because in principle there could be pressure at some point to say that money has to be earned now, so that you might be restricted in your therapy and treatment options. (Interviewee 3, 56 years.)*

The reasoning behind this occurrence is rooted in the profit-based orientation of PE companies, which is assumed to influence medical decisions in providing only or more profitable services [41,42]. Some practice-oriented German articles even claim that sales pressure is put on physicians under PE ownership [15,43]. Here, the concern is focused on physician treatment recommendations, which might shift towards more invasive treatments due to profitability reasons [40,44]. This underlines the conflict of interest that is expected to occur. In this regard, Moses et al. [39] argue that in the outpatient setting, physician autonomy has not yet been diminished by profitability pressures as it is in the inpatient setting.

Whereas the arguments above circulate around the reduction of physician autonomy, authors also reason for autonomy preservation. They argue that a PE ownership structure allows physicians to retain a higher degree of autonomy compared to hospital groups in the US [3,45,46].

#### 3.2.2. Impact on Quality of Care

Under PE involvement, the common opinion that ‘economics must serve the goals of medicine—and not vice versa’ [47] (p. A1692) is called into question. Indeed, some concerns are linked to the belief that with PE involvement, quality of care (QoC) is no longer the highest priority, as it is substituted by the profit interests of PE companies [8,11,13,14,37,40,45,48,49,50,51,52]. Here, a close relation to autonomy concerns is observable because both are frequently combined in the argumentative constructs. It is argued that the profit orientation of PE companies stands in conflict with medical principles and consequently affects the QoC in a negative way [8,14,42]. For example, patients with more complex diagnoses who might require more costly health care might be more likely to be rejected [4,41,45]. A similar argument as in the prior section is seen, where the concern is focused on prioritizing profitable treatments and patients. This ‘cherry picking’ of lucrative services is corroborated by Allroggen [42], showing that dental medical care centres owned by for-profit oriented investors (not only PE companies), on average, bill more profitable services.

In addition, among dermatologists in the US, a common concern is expressed that less-qualified personnel are employed among PE-owned practices instead of expensive and scarce physicians to reduce costs [4,13,40]. Likewise, Allroggen [42] refers to an incident in Spain where patients were treated by unqualified personnel with low-quality materials in an investor-owned dental practice group that then had to be shut down by the state.

The perception that with PE entering the outpatient health care sector, the focus will shift from patients towards financial profits is mirrored in the interviews. PE involvement in medical practice is expected to generate profit-oriented pressures that might restrict the autonomy of physicians in their treatment choices. Despite arguing that treatment decisions should be made from necessity and not economic considerations, interviewees expect pressure to sell profitable services affecting their treatment choices:

*But I leave the decision up to him (patient), now that I have the freedom. But if I had economic guidelines or economic pressure on my neck, then I would probably tend more towards turnover and sell that to him. (Interviewee 10, 50 years.)*

Contrary to the perception that quality of care is negatively impacted by PE involvement, other authors argue that PE involvement actually serves as an enabler; with managerial professionals from the PE company taking over administrative and bureaucratic tasks, these tasks can be performed more efficiently, and physicians can focus more on patient care. These possible advantages were complemented in the interviews by the possibility of better branding and marketing with PE involvement, a perception that was primarily mentioned by the younger interview participants.

#### 3.2.3. Impact on Work-Life Balance

The literature acknowledges that PE companies are entering the market during a change that is in their favour; O’Donnell et al. [6] and Francis et al. [44] describe the trend that many self-employed physicians from the baby-boomer generation in the US are facing retirement and are struggling to find successors due to generational differences between them and younger-generation physicians [53]. Similar generational differences are identifiable in Germany [15,28,42]. The younger generation places less priority on their medical self-employment and more on their work-life balance [4,44,53]. Correspondingly, DeWane et al. [4] and Ennenbach [27] describe a desire of the younger generation to work in teams with less hierarchical structures. Furthermore, increasing regulatory and administrative burdens hinder younger physicians from becoming self-employed [27,42].

The tendencies to avoid the risks associated with self-employment, to place a higher priority on a sufficient work-life balance, and the desire for team structures and other advantages of employment, was supported by younger interviewees:

*I realized that I would rather work in a large team and with many colleagues. […] It’s the responsibility. You don’t just have your own existence that you carry, but many other existences in self-employment. […] And that doesn’t end on Friday afternoon at 3 pm. That’s when it probably starts with all the accounting, stuff and taxes, I don’t know what would probably be omitted, which you don’t learn in your studies. (Interviewee 8, 25 years.)*

In the literature, the generational differences whereby the younger generation of physicians places less priority on their medical self-employment but on their work-life balance is presented as an opportunity for PE companies [4,44,53]. Gilreath et al. [5] underline this view, as PE structures can offer those benefits to physicians. Laschet [54] argues that young physicians can choose the environment in which they want to work. With increasing demands for young physicians [55], companies may have to agree to provisions for medical autonomy in contractual agreements. Allroggen describes a greater wish for team structures and lower hierarchies among younger physicians [42].

On a related note, interviewees argued that PE employment structures such as flexible working times, parental leave options, a sufficient salary and additional corporate benefits provide personal autonomy. With self-employed physicians experiencing difficulties in finding successors, one self-employed physician even described the PE working models as clearly more ‘future-oriented’. The interviews also underline the argument of generational differences. Younger physicians put more emphasis on work-life balance while their older peers placed more value on economic success.

*I don’t want to take out a loan of 800,000 or a million euros to buy a practice that I’ll have to pay off for the next 50 years because I don’t want to work that long. (Interviewee 14, 35 years* *.)*

#### 3.2.4. Impact on Sustainability

Fueled by the short investment horizon of PE companies [2,13], physicians expressed concerns regarding the sustainability of PE-owned physician practices [11,27,37,40,42,56]. Practices are targeted only for a short period of time, while the real return results from the resale of the practice or practice group, resulting in a short-term strategic focus [56]. Furthermore, other authors claim that with the quick exit of a PE company, the organizational structure and sustainability of the respective practices are disturbed [37,40,57]. This relates to the PE ‘buy-and-build’ strategy, i.e., the consolidation of small practices into a larger consortium in a fragmented market because it enables quick growth prior to the company’s exit [2,8]. Once more, a close connection to the prior section regarding the profit maximization interest of PE companies is observable [40].

A specific concern relates to the compensation prices paid by PE companies for physician practices. It is claimed that these prices are often far above the regular market prices [58,59]. This alienates potential younger successors, as they are not able to pay similar prices [8,54]

At the same time, these attractive compensation prices might be beneficial opportunities for retiring physicians [6]. Thus, the potential of a high pay-out by a PE company when retiring is attractive to them despite their negative perceptions [14]. Or, as an interviewee puts it:

*Actually, I think you guys [PE] are crap, but if you pay me that much money then you can have it.’ (Interviewee 10, 50 years.)*

Concerns related to the geographical areas targeted by PE were also expressed, since infrastructure needs to allow for quick growth realization [2,37]. Bruch et al. [60] and Appelbaum and Batt [2] found that PE-backed practices are primarily located in urban or suburban areas with above-average median household income in the US. Haaß et al. [8] and Laschet [54] present similar findings for Germany. This might have a cost-driving effect in those areas, while rural regions struggle to sustain sufficient health care accessibility [2,8,45].

On the other hand, the investment capital offered by PE companies is seen as an opportunity, especially in specialties that require expensive equipment, such as dentistry [5,33,46,54,61,62]. In this context, PE companies are considered to be value-adding entities, as they provide capital for investments in new equipment and streamline processes to deliver high-quality care more efficiently [5,41,46,61]. Among the interviewees, however, there was no consensus as to whether PE investment capital is considered a significant advantage. The extent to which new technologies are necessary to improve QoC was questioned, predominantly by older and self-employed physicians.

#### 3.2.5. Lack of Medical vs. Managerial Expertise

Interviewees indicated that PE companies lack the medical expertise that is necessary for successful collaboration and operational decision-making. Likewise, the short investment horizon in which PE companies act was seen as a key hindering factor for strategic planning.

Whereas the PE’s lack of medical expertise was posed as a concern, interviewees presented their own insufficient managerial and administrative knowledge as a supportive argument for PE companies. Insufficient knowledge about the managerial and administrative dimension of medicine led to a certain insecurity for younger physicians. They described the inadequate preparation in the studies:

*So, considering this entrepreneurial activity and founding a practice, you might have two lectures, but you don’t really know anything about it.’ (Interviewee 9, 25 years.)*

One young interviewee even described the financial aspects of medicine as a ‘red rag’, which is not being talked about or discussed, resulting in a reality shock for young physicians entering work after university. These young physicians had to acquire managerial and administrative knowledge on their own.

#### 3.2.6. Taxation Issues

Furthermore, a societal concern was presented by the argument that PE companies remove profits from the health care system [63]. Scheuplein et al. [63] investigated PE involvement in the German health care sector and reported that a large number of PE companies have their headquarters in tax havens such as the Cayman Islands. Thus, profits generated in the German health care system are not being taxed in Germany but are allocated to investors and removed from the health care system [15,27,43,54,64,65,66,67,68]. The removal of profits from the health care system was also criticised by the interviewees. The fact that PE companies remove profits from the health care system by paying out returns to their investors and having their headquarters located in tax havens was considered unjustifiable.

*I actually find that rather reprehensible, because a large part of the money is more or less provided by our social system and that is a bit like plundering the state.’ (Interviewee 2, 53 years.)*

## 4. Discussion

This explorative study set out to elucidate physicians’ perspectives on PE companies entering the outpatient health care setting. The prior section has shown a complex network of overlapping and intertwined arguments regarding physicians’ concerns and potential opportunities with the involvement of PE companies in outpatient health care settings. Notably, the predominant perception was of a critical nature. Physicians feared the demise of their professional medical and self-employed managerial autonomy [27,28,29,30,31,32,33,34,35,36,37] and subsequently a potential deterioration of quality of care due to profit-oriented treatment choices. Thereby, an ideal image of the self-determined medical profession shapes the physicians and builds a root cause for their concerns when they encounter external economic interests in their profession [27,69]. This concern is part of an ongoing discussion between economics on one end and physicians on the other [69,70], and shows the potential conflict between the economic interests of the PE company and the medical interests of the physicians.

At the same time, the results also indicate potential opportunities for physicians. With PE involvement allowing for a more efficient division of labour, physicians can focus on their medical work and might experience a considerable reduction of workload due to less administrative work, potentially resulting in better work-life balance. These potential benefits were perceived particularly among younger interviewees, perhaps because perceptions of and desires for their work have changed over time. Due to the increasing importance of a stricter separation between work and private life, younger physicians potentially attribute greater importance to the possible relief from administrative support through PE involvement than older physicians. This change in attitude among younger physicians to no longer subordinate their lives solely to professional obligations was also stressed by older interviewees and has been cited as a prime reason for struggles with finding a successor. Additionally, the financial risk for a practice takeover is perceived as a critical burden that could make younger physicians more willing to work in PE-owned outpatient practices instead of being self-employed. These developments could considerably promote the scope and scale of PE companies in the outpatient sector.

The insights generated by this study need to be considered in light of some limitations. First, screening and coding were conducted by one author only. Despite regular discussions and reflections about the analysis process, we thus cannot rule out false exclusions in the review process or omissions of perceptions beyond the identified themes. Second, interviewees were selected using criteria-based sampling. However, a substantial number of potential interviewees refrained from responding to the interview invite and the reasons for nonresponse remain unknown. As such, the sample could reflect selective opinions and the first indications of generational differences in how physicians perceive PE involvement are potentially overestimated. Third, we deliberately decided to include non-peer reviewed references because we intended to capture physicians’ perceptions expressed in different formats. The perceptions identified in this study could therefore be driven by the substantial number of opinion papers identified in the review. At the same time, the choice that we made acknowledges that perceptions are not only shaped by peer reviewed empirical studies but also through other means.

Despite these limitations, the results imply that the discussion surrounding PE involvement in outpatient care needs to be conducted in a more nuanced and differentiated manner. As mentioned by many authors, the arguments identified in the scientific literature often lack sufficient empirical support [6,11,14,38,39,60,71,72,73], especially concerning the critical arguments [13,14,37,49,54,74]. The debate is often led by single negative incidents overarching the number of successfully operating practices that are backed by PE companies [42,72]. Anecdotal evidence, frequently repeated and picked up by professional outlets, therefore played a strong role in shaping physicians’ perceptions.

The lack of empirical evidence limits the validity of the identified arguments and underlines the early stage of this research field as well as the strong necessity for further research concerning the impact of PE involvement on, for example, quality of care [11,14,60]. Thus, longitudinal studies such as the work conducted in the nursing sector by Pradhan et al. [75] and Gupta et al. [76] are equally necessary in the outpatient setting. However, conducting such research also requires more transparency. To date, detailed public reporting about PE transactions in outpatient care is limited, and most transactions happen under the radar [1]. To verify the different arguments empirically and enable informed decision-making, transparency and evidence are needed.

## 5. Conclusions

This paper shows the scope of unanswered questions in the emerging field of PE involvement in outpatient health care. Sufficient transparency about PE involvement in the outpatient health care setting as well as long-term evidence on the effects of PE involvement in outpatient health care is needed. Thereby, evidence measuring the effects on physician wellbeing, the quality of care delivered, and health care expenditure will be of immense importance for a factual discussion. Future research providing this evidence is thus called for.

## Data Availability

The data presented in this study are available on request from the corresponding author.

## Supplementary Materials

The following supporting information can be downloaded at: https://www.mdpi.com/article/10.3390/ijerph192315480/s1, Overview of the literature results, interview participants, and coding tree.
