# Supplementary material for: Physicians’ Perspectives Regarding Private Equity Transactions in Outpatient Health Care—A Scoping Review and Qualitative Analysis
Source: Int J Environ Res Public Health. 2022 Nov 22;19(23):15480. doi: 10.3390/ijerph192315480 (PMC9737937; doi:10.3390/ijerph192315480)
Supplement: Supplementary file 1 [file ijerph-19-15480-s001.zip › ijerph-1959606-supplementary.pdf]

Physicians' perspectives regarding private equity transactions in outpatient health care – a scoping review and qualitative analysis

Tim N. Nolte [1], Felix Miedaner [2] and Sandra Sülz [1,*]

# - Supplementary Material -

Characteristics of interview participants

**Individual characteristics**	**N (*Mean*)**	**% (*SD*)**
Age	*40.0*	*12.4*
Practicing time	*12.5*	*10.6*
Gender		
Male	12	85.7
Female	2	14.3
Specialty		
Dentistry	9	64.3
Oral and maxillofacial surgery	2	14.3
Dermatology	1	7.1
Paediatrics	1	7.1
Ophtalmology	1	7.1
Organization		
Group practice	9	64.3
Solo practice	2	14.3
University hospital, outpatient care	2	14.3
Practice group	1	7.1
Employment status		
Self-employed	6	42.9
Employed	8	57.1
Knowledge about PE		
Yes	12	85.7
No	2	14.3
Contact to PE		
Yes	4	28.6
No	10	71.4

SD: standard deviation; Means and standard deviations are displayed in italic; Due to rounding, percentages may not add up to 100%. Group practice (Gemeinschaftspraxis) and practice group (Praxisgemeinschaft) are different cooperation forms in the German outpatient sector.

Coding Tree

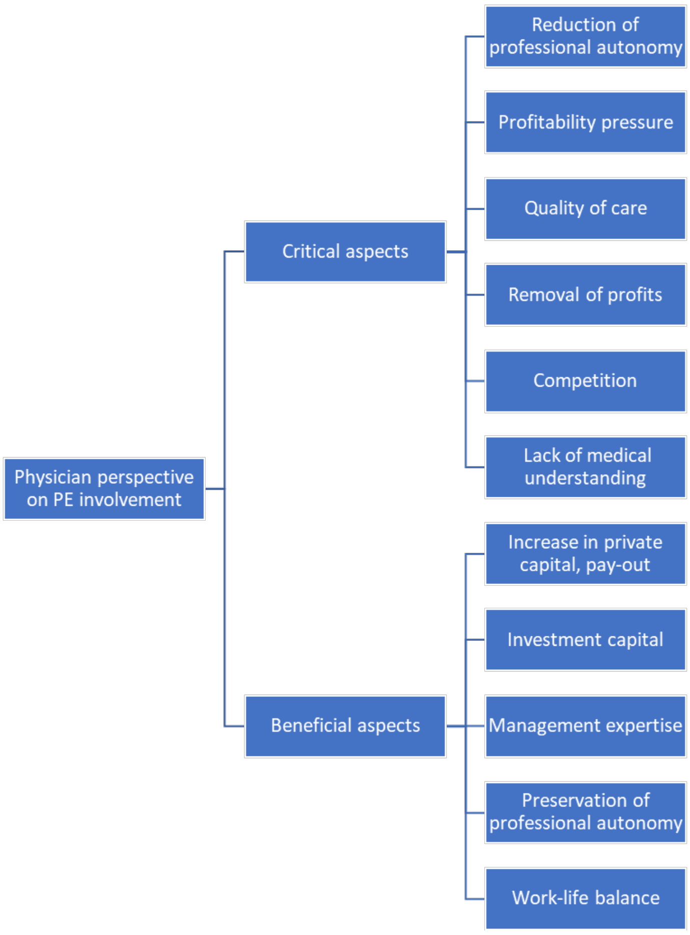
Physician perspective on PE involvement
Critical aspects
Reduction of professional autonomy
Profitability pressure
Quality of care
Removal of profits
Competition
Lack of medical understanding
Beneficial aspects
Increase in private capital, pay-out
Investment capital
Management expertise
Preservation of professional autonomy
Work-life balance

Literature Table

References	Physician autonomy	Quality of care	Work-life-balance	Sustainability	Lack of expertise	Taxation
aerztblatt.de. Zahnärzte warnen vor MVZ-Übernahmen durch Kapitalinvestoren. *Dtsch. Ärzteblatt* **2018**,. Available online: https://www.aerzteblatt.de/treffer?mode=s&wo=1041&typ=1&nid=99030&s=Private&s=equity (accessed on 15 November 2022).	x	x			x	
aerztblatt.de. Wir kennen keinen Fall, in dem Kapitalinteressen die ärztliche Entscheidung infrage gestellt hätten. *Dtsch. Ärzteblatt* **2018**,. Available online: https://www.aerzteblatt.de/treffer?mode=s&wo=1041&typ=1&nid=98076&s=Private&s=equity (accessed on 15 November 2022).	x		x			
aerzteblatt.de. Gesundheitssektor zieht Investoren an. Deutsches Ärzteblatt. 2019. Available online: https://www.aerzteblatt.de/treffer?mode=s&wo=1041&typ=1&nid=102620&s=Private&s=equity (accessed on 15 November 2022).		x				x
aerzteblatt.de. Hartmannbund ruft zum Dialog über demografischen Wandel bei Ärzten auf. Deutsches Ärzteblatt. 2019. Available online: https://www.aerzteblatt.de/treffer?mode=s&wo=1041&typ=1&nid=107278&s=hartmannbund&s=ruft (accessed on 15 November 2022).		x				
aerzteblatt.de. Union hat keine Bedenken bei Beteiligungen Renditeorientierter Kapitalanleger an MVZ. Deutsches Ärzteblatt. 2019. Available online: https://www.aerzteblatt.de/treffer?mode=s&wo=1041&typ=1&nid=107370&s=Kapitalanleger&s=MVZ &s=Union (accessed on 15 November 2022).		x		x		
aerzteblatt.de. Gesundheitsexperten Stehen Fremdinvestoren Überwiegend Kritisch Gegenüber. Deutsches Ärzteblatt. 2020. Available online: https://www.aerzteblatt.de/treffer?mode=s&wo=&typ=1&nid=110813&s=Gesundheitsexperten&s= fremdinvestoren&s=kritisch (accessed on 15 November 2022).		x		x		
Allroggen, S. Veränderung der Zahnmedizinischen Versorgungsstruktur aus Sicht einer Kassenzahnärztlichen Vereinigung. *MKG Chir.* **2020**, *13*, 10–19. https://doi.org/10.1007/s12285-019-00236-3.	x	x	x	x		
Appelbaum, E.; Batt, R. *Private Equity Buyouts in Healthcare: Who Wins, Who Loses?*; Institute for New Economic Thinking Working: New York, NY, USA, 2020. https://doi.org/10.36687/inetwp118.	x	x		x		
arzt-wirtschaft.de. Pflegeheime und Praxen: Finanzinvestoren Greifen nach der Gesundheitsbranche. A&W Online 2019. Available online: https://www.arzt-wirtschaft.de/finanzen/geldanlagen/pflegeheime-und-praxen-finanzinvestoren-greifen-nach-der-gesundheitsbranche/ (accessed on 15 November 2022).				x		x
Arzt-wirtschaft.de. Bundeszahnärztekammer fordert Stopp von Fremdkapital in der Zahnmedizin. A&W Online. 2020. Available online: https://www.arzt-wirtschaft.de/finanzen/honorare/bundeszahnaerztekammer-fordert-stopp-von-fremdkapital-in-der-zahnmedizin/ (accessed on 15 November 2022).	x	x		x		x
Bennett, R.G. Further thoughts on dermatology and equity-owned dermatology practices. *J. Am. Acad. Dermatol.* **2019**, *81*, e11–e12. https://doi.org/10.1016/j.jaad.2018.11.055.		x				

References	Themes					
	Physician autonomy	Quality of care	Work-life-balance	Sustainability	Lack of expertise	Taxation
Bennett, R.G. Conflicts of interest for physician owners of private equity—Owned medical practices. *J. Am. Acad. Dermatol.* **2020**, *82*, e35. https://doi.org/10.1016/j.jaad.2019.09.033.		x				
Bobsin, R. *Finanzinvestoren in der Gesundheitsversorgung in Deutschland: 20 Jahre Private Equity—Eine Bestandsaufnahme*, 4th ed.; Offizin-Verlag: Hannover, Germany, 2019.	x	x		x		x
Bruch, J.D.; Borsa, A.; Song, Z.; Richardson, S.S. Expansion of Private Equity Involvement in Women's Health Care. *JAMA Intern. Med.* **2020**, *180*, 1542–1545. https://doi.org/10.1001/jamainternmed.2020.3567.		x		x	x	
Casalino, L.P.; Saiani, R.; Bhidya, S.; Khullar, D.; O'Donnell, E. Private equity acquisition of physician practices. *Ann. Intern. Med.* **2019**, *170*, 114–115. https://doi.org/10.7326/M18-2363.	x	x			x	
DeCamp, M.; Sulmasy, L.S. Ethical and Professionalism Implications of Physician Employment and Health Care Business Practices: A Policy Paper From the American College of Physicians. *Ann. Intern. Med.* **2021**, *174*, 844–851. https://doi.org/10.7326/m20-7093.	x	x		x	x	
DeWane, M.E.; Mostow, E.; Grant-Kels, J.M. The corporatization of care in academic dermatology. *Clin. Dermatol.* **2020**, *38*, 289–295. https://doi.org/10.1016/j.clindermatol.2020.02.003.	x	x		x	x	
Engel, P. Profit auf Kosten der Patientinnen und Patienten? *MKG Chir.* **2020**, *13*, 6–9. https://doi.org/10.1007/s12285-019-00230-9.	x	x	x	x		x
Ennenbach, R. Ist Private Equity für die ambulante Versorgung notwendig, ein Fortschritt oder eine Gefahr? *MKG Chir.* **2020**, *13*, 3–5. https://doi.org/10.1007/s12285-020-00241-x.	x	x	x	x		x
Francis, J.; Konda, S.; Motaparthi, K.; Grant-Kels, J.M. Response to letter to the editor from Bennett and further thoughts on the corporatization of dermatology and private equity–backed dermatology groups. *J. Am. Acad. Dermatol.* **2019**, *81*, e13–e14. https://doi.org/10.1016/j.jaad.2019.03.024.	x	x		x		
Gilreath, M.; Patel, N.C.; Suh, J.; Brill, J.V. Gastroenterology Physician Practice Management and Private Equity: Thriving in Uncertain Times. *Clin. Gastroenterol. Hepatol.* **2021**, *19*, 1084–1087. https://doi.org/10.1016/j.cgh.2021.03.015.	x			x	x	
Glöser, S. Gesundheitswesen wird für Investoren immer attraktiver. *Dtsch. Ärzteblatt* **2019**, *116*, 4. Available online: https://www.aerzteblatt.de/treffer?mode=s&wo=1041&typ=16&aid=206759&s=Gesundheitswesen&s=Investoren&s=attraktiver&s=f%FCr&s=immer&s=wird (accessed on 15 November 2022).				x		x
Gondi, S.; Song, Z. Private Equity Investment in Health Care. *JAMA J. Am. Med. Assoc.* **2019**, *322*, 468–469. https://doi.org/10.1001/jama.2019.7844.		x			x	
Gondi, S.; Song, Z. Potential Implications of Private Equity Investments in Health Care Delivery. *JAMA J. Am. Med. Assoc.* **2019**, *321*, 1047–1048. https://doi.org/10.1001/jama.2019.1077.		x		x	x	
Haaß, F.A.; Ochmann, R.; Julia, G.; Albrecht, M.; Nolting, H.-D. *Investorenbetriebene MVZ in der Vertragszahnärztlichen Versorgung: Entwicklung und Auswirkungen*; Duncker Humblot GmbH: Berlin, Germany, 2021.	x	x		x		

References	Themes					
	Physician autonomy	Quality of care	Work-life-balance	Sustainability	Lack of expertise	Taxation
Hilienhof, A. Gesundheitsmarkt: Finanzinvestoren auf dem Vormarsch. *Dtsch. Ärzteblatt* **2019**, *116*, A340. Available online: https://www.aerzteblatt.de/treffer?mode=s&wo=1041&typ=16&aid=205710&s=Investoren&s=Vormarsch&s=auf&s=dem (accessed on 15 November 2022).				x		x
Hsu, A.; Kohli, N. YPS Report: The Role of Private Equity in Medicine. *Mo. Med.* **2018**, *115*, 333. Available online: http://www.ncbi.nlm.nih.gov/pubmed/30228757 (accessed on 15 November 2022).	x	x		x		x
Khetpal, S.; Lopez, J.; Steinbacher, D.M. Trends in Private Equity Deals in Oral and Maxillofacial Surgery and Dentistry. *J. Oral Maxillofac. Surg.* **2021**, *79*, 513–515. https://doi.org/10.1016/j.joms.2020.10.010.	x	x			x	
Kirsh, G.M.; Kapoor, D.A. Private Equity and Urology: An Emerging Model for Independent Practice. *Urol. Clin. N. Am.* **2021**, *48*, 233–244. https://doi.org/10.1016/j.ucl.2020.12.004.				x	x	
Konda, S.; Francis, J. The evolution of private equity in dermatology. *Clin. Dermatol.* **2020**, *38*, 275–283. https://doi.org/10.1016/j.clindermatol.2020.02.007.	x	x		x	x	
Konda, S.; Francis, J.; Motaparthi, K.; Grant-Kels, J.M. Future considerations for clinical dermatology in the setting of 21st century American policy reform: Corporatization and the rise of private equity in dermatology. *J. Am. Acad. Dermatol.* **2019**, *81*, 287–296.e8. https://doi.org/10.1016/j.jaad.2018.09.052.		x		x	x	
Korzilius, H. Ambulante Versorgung: Investoren auf Einkaufstour. *Dtsch. Ärzteblatt* **2018**, *115*, A1688–1692. Available online: https://www.aerzteblatt.de/archiv/201014/Ambulante-Versorgung-Investoren-auf-Einkaufstour (accessed on 15 November 2022).		x	x	x		
Laschet, H. Investoren im Gesundheitswesen: Plage oder Partner fürs Gemeinwohl? Ärzte Zeitung. 2020. Available online: https://www.aerztezeitung.de/Politik/Investoren-im-Gesundheitswesen-Plage-oder-Partner-fuers-Gemeinwohl-413318.html (accessed on 15 November 2022).				x		x
Laschet, H. Versorgung: Private Equity ist auch eine Chance. Ärzte Zeitung. 2021. Available online: https://www.aerztezeitung.de/Politik/Private-Equity-ist-auch-eine-Chance-417854.html (accessed on 15 November 2022).	x	x	x	x		x
Laschet, H. Finanzierung: Private Equity in MVZ? Mehr Evidenz täte der Debatte gut. Ärzte Zeitung. 2021. Available online: https://www.aerztezeitung.de/Wirtschaft/Private-Equity-in-MVZ-Mehr-Evidenz-taete-der-Debatte-gut-419020.html (accessed on 15 November 2022).		x	x	x		
Lundy, D.W. A Day at the Office: Private Practice and Private Equity. *Clin. Orthop. Relat. Res.* **2019**, *477*, 955–957. https://doi.org/10.1097/CORR.0000000000000758.		x		x	x	
Maibach-Nagel, E. Fremdinvestoren im Gesundheitssystem: Ungesunder Wettbewerb. *Dtsch. Ärzteblatt* **2018**, *115*, A-1675. Available online: https://www.aerzteblatt.de/archiv/201003/Fremdinvestoren-im-Gesundheitssystem-Ungesunder-Wettbewerb (accessed on 15 November 2022).				x	x	
Miller, L.E.; Rathi, V.K.; Naunheim, M.R. Implications of Private Equity Acquisition of Otolaryngology Physician Practices. *JAMA Otolaryngol. Head Neck Surg.* **2020**, *146*, 97–98. https://doi.org/10.1001/jamaoto.2019.3738.	x	x	x	x	x	

References	Themes					
	Physician autonomy	Quality of care	Work-life-balance	Sustainability	Lack of expertise	Taxation
Montgomery, U.F. Investoren in der ambulanten Versorgung: Erst der Patient, dann die Ökonomie. *Dtsch Ärzteblatt* **2018**, *115*, A1692. Available online: https://www.aerzteblatt.de/treffer?mode=s&wo=1041&typ=16&aid=201048&s=Private&s=equity (accessed on 15 November 2022).		x		x		x
Moses, M.J.; Weiser, L.G.; Bosco, J.A. The Corporate Practice of Medicine. *J. Bone Joint Surg.* **2020**, *102*, e53. https://doi.org/10.2106/JBJS.19.01404.	x	x		x		
Novice, T.; Portney, D.; Eshaq, M. Dermatology resident perspectives on practice ownership structures and private equity-backed group practices. *Clin. Dermatol.* **2020**, *38*, 296–302. https://doi.org/10.1016/j.clindermatol.2020.02.008.	x	x		x		
O'Donnell, E.M.; Lelli, G.J.; Bhidya, S.; Casalino, L.P. The Growth of Private Equity Investment in Health Care: Perspectives from Ophthalmology. *Health Affairs* **2020**, *39*, 1026–1031. https://doi.org/10.1377/hlthaff.2019.01419.	x	x		x	x	
Osterloh, F. Patientenversorgung unter Druck: Gegen die Kommerzialisierung. Deutsches Ärzteblatt, 116. 2019. Available online: https://www.aerzteblatt.de/archiv/207864/Patientenversorgung-unter-Druck-Gegen-die-Kommerzialisierung (accessed on 15 November 2022).		x				x
Patel, N.A.; Afshar, S. Implications of Private Equity in Oral and Maxillofacial Surgery. *J. Oral Maxillofac. Surg.* **2020**, *78*, 1456–1458. https://doi.org/10.1016/j.joms.2020.03.046.	x	x		x		
Patel, N.S.; Groth, S.; Sternberg, P. The Emergence of Private Equity in Ophthalmology. *J. Am. Acad. Dermatol.* **2019**, *137*, 601–602. https://doi.org/10.1001/jamaophthalmol.2019.0964.	x	x	x	x	x	
Reddy, R. Private Equity Investments in Women's Health and Obstetrics and Gynecology Practices. *Obstet. Gynecol.* **2020**, *136*, 1217–1220. https://doi.org/10.1097/AOG.0000000000004151.	x	x	x	x	x	x
Resneck, J.S.; Philip, R.L. Dermatology practice consolidation fueled by private equity investment potential consequences for the specialty and patients. *JAMA Dermatol.* **2018**, *154*, 13–14. https://doi.org/10.1001/jamadermatol.2017.5558.	x	x		x	x	x
Resnick, M.J. Re: The growth of private equity investment in health care: Perspectives from ophthalmology. *J. Urol.* **2020**, *204*, 1371. https://doi.org/10.1097/JU.0000000000001278.02.		x		x	x	
Satiani, B.; Zigrang, T.A.; Bailey-Wheaton, J.L. Should surgeons consider partnering with private equity investors? *Am. J. Surg.* **2020**, *222*, 453–458. https://doi.org/10.1016/j.amjsurg.2020.12.028.	x	x			x	x
Scheffler, R.M.; Alexander, L.M.; Godwin, J.R. Soaring Private Equity Investment in the Healthcare Sector: Consolidation Accelerated, Competition Undermined, and Patients at Risk. 2021. Available online: https://publichealth.berkeley.edu/wp-content/uploads/2021/05/Private-Equity-I-Healthcare-Report-FINAL.pdf (accessed on 15 November 2022).	x	x		x	x	x
Scheuplein, C.; Evans, L.M.; Merkel, S. *Übernahmen durch Private Equity in Deutschen Gesundheitssektor: Eine Zwischenbilanz für die Jahre 2013 bis 2018*; IAT: Gelsenkirchen, Germany, 2019. https://doi.org/http://dx.doi.org/10.13140/RG.2.2.30503.19366. IAT Discussion Paper No. 19/01.				x		x
Skaljic, M.; Lipoff, J.B. Association of private equity ownership with increased employment of advanced practice professionals in outpatient dermatology offices. *J. Am. Acad. Dermatol.* **2021**, *84*, 1178–1180. https://doi.org/10.1016/j.jaad.2020.05.024.		x		x		

References	Themes					
	**Physician autonomy**	**Quality of care**	**Work-life-balance**	**Sustainability**	**Lack of expertise**	**Taxation**
Springer Medizin. Die Gesichter der dentalen Ketten. *Junge Zahnarzt* **2019**, *10*, 24–33. https://doi.org/10.1007/s13279-019-0044-x.	x	x	x	x		
Tan, S.; Seiger, K.; Renehan, P.; Mostaghimi, A. Trends in Private Equity Acquisition of Dermatology Practices in the United States. *JAMA Dermatol.* **2019**, *155*, 1013–1021. https://doi.org/10.1001/jamadermatol.2019.1634.	x	x		x	x	
Waldmann, R.; Kelsey, A.; Grant-Kels, J.M. Comment on: Conflicts of interest for physician owners of private equity–owned medical practices. *J. Am. Acad. Dermatol.* **2020**, *82*, e33. https://doi.org/10.1016/j.jaad.2019.08.087.		x				
Wasner, A. Wie stark werden sich Private-Equity- Übernahmen auf die ambulante Versorgung auswirken? Medical Tribune. 2019. Available online: https://www.medical-tribune.de/praxis-und-wirtschaft/niederlassung-und-kooperation/artikel/wie-stark-werden-sich-private-equity-uebernahmen-auf-die-ambulante-versorgung-auswirken/ (accessed on 15 November 2022).		x		x		x
Wasner, A. Zahl der MVZ in Private-Equity-Besitz auch 2020 gestiegen. Medical Tribune. 2021. Available online: https://www.medical-tribune.de/praxis-und-wirtschaft/niederlassung-und-kooperation/artikel/zahl-der-mvz-in-private-equity-besitz-auch-2020-gestiegen/ (accessed on 15 November 2022).		x		x		
Zhu, J.M.; Polsky, D. Private Equity and Physician Medical Practices—Navigating a Changing Ecosystem. *N. Engl. J. Med.* **2021**, *384*, 981–983. https://doi.org/10.1056/NEJMp2032115.	x	x		x	x	
Zhu, J.M.; Hua, L.M.; Polsky, D. Private Equity Acquisitions of Physician Medical Groups Across Specialties, 2013-2016. *JAMA J. Am. Med. Assoc.* **2020**, *323*, 663–665. https://doi.org/10.1097/JU.0000000000001215.		x				
